# Green tea extract induces genes related to browning of white adipose tissue and limits weight-gain in high energy diet-fed rat

**DOI:** 10.1080/16546628.2017.1347480

**Published:** 2017-07-14

**Authors:** Li-Han Chen, Yi-Wen Chien, Chung-Tiang Liang, Ching-Hung Chan, Meng-Han Fan, Hui-Yu Huang

**Affiliations:** ^a^ YongLin Biomedical Engineering Center, National Taiwan University, Taipei City, Taiwan; ^b^ School of Nutrition and Health Sciences, Taipei Medical University, Taipei City, Taiwan; ^c^ National Laboratory Animal Center, Taipei City, Taiwan; ^d^ Department of Food Science, Nutrition, and Nutraceutical Biotechnology, Shih Chien University, Taipei City, Taiwan

**Keywords:** Anti-obesity, beige adipose tissue, browning of white adipose tissue, green tea extract, pathways of browning

## Abstract

**Background:** A wealth of research has reported on the anti-obesity effects of green tea extract (GTE). Although browning of white adipose tissue (WAT) has been reported to attenuate obesity, no study has disclosed the effects of GTE on browning in Sprague Dawley rats.

**Objectives:** The aims of the study were to investigate the effects of GTE on anti-obesity and browning, and their underlying mechanisms.

**Methods:** Four groups of rats (n=10/group) were used including a normal diet with vehicle treatment, and a high-energy diet (HED) with vehicle or GTE by oral gavage at 77.5 or 155 mg/kg/day for 8 weeks. Body weight, fat accumulation, and serum biochemical parameters were used to evaluate obesity. The gene expressions were analyzed using RT-qPCR and western blotting.

**Results:** GTE modulated HED-induced body weight, fat accumulation, and serum levels of triacylglycerol, total cholesterol, low-density lipoprotein, free fatty acids, aspartate aminotransferase, and alanine aminotransferase. Moreover, GTE enhanced the serum high-density lipoprotein. Most importantly, the biomarkers of beige adipose tissue were up-regulated in WAT in GTE-given groups. GTE induced genes involved in different pathways of browning, and reduced transducin-like enhancer protein-3 in WAT.

**Conclusion:** Our results suggest that GTE may improve obesity through inducing browning in HED-fed rats.

**Abbreviations**: ALT: Alanine transaminase; AST: Aspartate transaminase; BAT: Brown adipose tissue; BMP-7: Bone morphogenetic protein-7; BW: Body weight; CIDEA: Cell death activator; CPT-1: Carnitine palmitoyltransferase-1; EFP: Epididymal fat pad; FFA: Free fatty acid; FGF-21: Fibroblast growth factor-21; GTE: Green tea extract; HDL: High-density lipoprotein; HED: high-energy diet; LDL: Low-density lipoprotein; MFP: Mesenteric fat pad; PGC-1α: Activates PPAR-γ coactivator-1; PPAR-γ: Peroxisome proliferator-activated receptor-γ; PRDM-16: PR domain containing 16; RFP: Renal fat pad; SD: Sprague Dawley; TC: Total cholesterol; TG: Triacylglycerol; TLE-3: Transducin-like enhancer protein-3: UCP-1: Uncoupling protein-1; WAT: White adipose tissue.

## Introduction

Obesity is one of the major risk factors for pathological disorders, including diabetes, hypertension, atherosclerosis, and cancer [1,2]. The prevalence of obesity is estimated to increase by 7% and 10% among men and women, respectively, by 2020 [3]. Therefore, to resolve the issue of obesity is an important requirement for human health. Several studies reported that food and food ingredients are good targets for improving obesity disorders, leading to the investigation of their anti-obesity mechanisms [4–6]. For example, green tea and its extracts were suggested to reduce body weight (BW) through inducing apoptosis, inhibiting adipogenesis, preventing energy uptake, enhancing lipolysis, elevating fatty acid oxidation-related genes, and increasing energy expenditure [7,8]. However, the effect of green tea on the browning of white adipose tissues (WAT) of Sprague Dawley (SD) rats is still unreported.

Nowadays, three types of adipose tissue are known, including WAT, brown adipose tissue (BAT), and beige adipose tissue. WAT is optimized to store energy in large lipid droplets for later use [9], and the accumulation of body fat in WAT leads to both hypertrophy and hyperplasia of white adipocytes [10]. These changes are associated with obesity-related diseases such as type-2 diabetes and an inflammatory response [1,11]. In contrast, BAT generates energy mostly in the form of heat [12], and promoting BAT activities was reported to prevent obesity [13]. Therefore, BAT was suggested as a potential target of anti-obesity therapy [14]. Recently, increasing research has focused on the other type of adipose tissue, beige adipose tissue, because beige adipose tissue was also identified as another potential candidate for obesity treatment due to its capacity to burn glucose and fat to produce heat [15]. Beige adipose tissue, traditionally seen as WAT, is a new form of adipose tissue and can be transformed from WAT [16]. Although beige adipose tissue and WAT are derived from Pax7− and Myf5− precursor cells and express different gene signatures from BAT [17–19], beige adipose tissue is similar to BAT in that it has more mitochondria and higher expressions of thermogenesis and lipolysis genes [20].

Several genes of different pathways were suggested to regulate the browning of WAT. Peroxisome proliferator-activated receptor-γ (PPAR-γ) is the master transcriptional regulator of fat differentiation, and in concert with the PR domain containing-16 (PRDM-16) activates PPAR-γ coactivator-1α (PGC-1α) to induce brown-selective genes [21–23]. Browning is also driven by the pathways involving either fibroblast growth factor-21 (FGF-21) or bone morphogenetic protein-7 (BMP-7) [24,25]. An increase of FGF-21 in WAT stabilizes PGC-1α to enhance browning [24]. Moreover, EWS/YBX-1/BMP-7 pathway was suggested to mediate browning [25]. Conversely, transducin-like enhancer protein-3 (TLE-3), which suppresses brown-selective genes and induces white-selective genes, is involved in a negative regulation pathway of the browning process [26]. Differences between beige adipose tissue and WAT are observed in genes regulating them and also in mitochondrial and fatty acid oxidation-related genes. Compared to WAT, beige adipose tissue presents higher levels of uncoupling protein-1 (UCP-1), cell death activator (CIDEA), and carnitine palmitoyltransferase-1 (CPT-1) [18,27,28]. Enhancement of these genes transforms WAT into fat-oxidizing and energy-expending machines [29]. The aim of this study was to investigate the effects of green tea extract (GTE) on anti-obesity and browning of WAT in SD rats fed a high-energy diet (HED). Additionally, we analyzed the gene expressions following GTE treatment to evaluate GTE-induced pathways of browning of WAT. Therefore, the present study could demonstrate new insights into the anti-obesity mechanism induced by GTE.

## Materials and methods

### Animals and diets

Male SD rats (6 weeks old, ~215 g) were purchased from a local supplier (LASCO, Taipei, Taiwan) and singly housed during the study. Rats were given seven days to acclimate to their new environment under standard laboratory conditions (a 12/12-h light/dark cycle, 22 ~ 24°C, 40%~60% humidity). All rats were fed ad libitum with the diets purchased from a local supplier. Ten rats given a normal diet (see Table 1 for ingredient composition) comprised the control group (C). The other 30 rats were fed an HED (see Table 1 for ingredient composition) and randomly and equally divided into three groups, including an HE group (given the HED), a 1X group (given the HED and 77.5 mg/kg/day of GTE), and a 2X group (given the HED and 155 mg/kg/day of GTE). For humans, giving 750 mg/day of GTE was reported to protect against obesity [30]. The rat dose was converted from a human equivalent dose based on the body surface area by the following formula from the US Food and Drug Administration: assuming a human weight of 60 kg, the dose for rat is 750 mg/day÷60 kg × 6.2  =  77.5 mg/kg/day; the conversion coefficient of 6.2 was used to account for differences between rats and humans. The GTE powder (Hong-Zhong Biotechnology, Tainan, Taiwan) was produced from green tea by standard procedures with a certificate of the analytical methods, and contained 83.5% total catechins, 38.5% epigallocatechin gallate (EGCG), 96.6% total polyphenols, and 1.8% caffeine (analyzed by HPLC). GTE was dissolved in 1 ml of distilled water and orally administrated to the 1X and 2X groups by gavage at 09:00 every day for eight weeks, while 1 ml of distilled water was given to the C and HE groups instead. Food and water intake levels were recorded every day, and BWs were measured weekly. Animals were sacrificed by CO_2_ at the end of the eighth week. All animal experiments were performed in accordance with the protocol (IACUC-10,303) approved by the Institutional Animal Care and Use Committee (IACUC) of Shih Chien University.

**Table 1.** Ingredient composition of the diets fed to rats.

Ingredient composition	Normal diet	High-energy diet
Corn starch	46.23%	4.50%
Dextrin	15.38%	1.49%
Casein-vitamin free	13.89%	13.96%
Sucrose	9.92%	27.89%
Fructose	0.00%	19.91%
Powdered cellulose	4.96%	4.70%
Soybean Oil	3.97%	21.90%
AIN 93M Mineral Mix	3.47%	3.47%
AIN 93 Vitamin Mix	0.99%	0.99%
Choline bitartrate	0.23%	0.23%
L-Cystine	0.17%	0.17%
t-Butylhydroquinone	0.79%	0.79%
kcal/g	3.8	4.50

### Adipose tissues sampling

WATs from mesenteric, epididymal, and perirenal sites were collected, rinsed with PBS, and then weighed immediately. All samples were stored at −80°C until the beginning of analysis.

### Serum biochemical parameters

After eight hours of starving, rat blood was collected and serum was obtained by centrifugation at 3500 g for 10 minutes at 4°C. Plasma triacylglycerol (TG), total cholesterol (TC), low-density lipoprotein (LDL), high-density lipoprotein (HDL), alanine transaminase (ALT), and aspartate transaminase (AST) were measured using a Hitachi 7080 analyzer (Hitachi, Japan). The free fatty acid (FFA) concentration was determined by an enzymatic colorimetric assay according to the manual of a commercial kit (Boehringer Mannheim, Germany).

### Determination of the adipocyte size of WAT

Epididymal fat pads (EFPs) were fixed in 4% paraformaldehyde (pH 7.2) at 4°C for 16 hours. EFP samples were dehydrated in absolute ethanol, cleared in xylene, and then embedded in paraffin. After cutting the paraffin into 4-μm sections, sections were stained with Harris hematoxylin and counterstained with eosin, and digital images were taken under light microscopy (Nikon, Japan). To analyze the digital images, the area, approximate diameter, perimeter, and shape factor were measured in 10 adipocytes (five slides for each rat, 10 rats per group). The analysis of these results was performed using the Sigma ScanPro4 program (Sigma, USA).

### RNA extraction and quantitative RT-PCR (RT-qPCR)

RNA was extracted using an RNeasy Mini kit (Qiagen, Germany), and 500 ng of total RNA of each sample was reverse-transcribed with an iScript cDNA Synthesis Kit (Bio-Rad, USA) according to instructions of the manufacturer. A qPCR was performed in a MyiQ Single-Color Real-Time PCR Detection System (Bio-Rad), and sequences of gene-specific primers (Purigo, Taiwan) are shown in Table 2. β-actin was used as an internal control for normalizing messenger (m)RNA levels of the tested genes.

**Table 2.** Primer sequences used in the RT-qPCR.

Gene	Gene accession numbers	Forward primers	Reverse primers
PPAR-γ	NM_013124.3	GACCTCTCTGTGATGGATGAC	TCGCACTTTGGTATTCTTGGA
PRDM-16	NM_001291029.1	GCAGACCCTGTGGGAGTCCTGAAA	GCTCCCCTGTGTGTGTCCTCAGAT
BMP-7	NM_001191856.2	CGCTCCAAGACTCCAAAGAA	GGTCTCGGAAGCTAACATACAG
FGF-21	NM_130752.1	CAACAACCAGATGGAACTCTCTA	GGTACACATTGTATCCGTCCTT
PGC-1α	AY237127.1	GCCGGAGCAATCTGAGTTAT	GATCACCAAACAGCCGTAGA
TLE-3	NM_053400.1	GATAGGCAGATGGACAGACAAG	GAGAAGATGGAGCAGAGAAACC
UCP-1	NM_012682.2	AGGGTTTGCGCCTTCTTT	GGGACTTCATCAGCTCTTTCTT
CPT-1	NM_031559.2	CGGAGCCAGGAGATATAGATAGA	GAATCTGACTGGGTGGGATTAG
CIDEA	NM_001170467.1	GGACACAGAGGAGTTCTTTCAG	CGAAGGTGACTCTGGCTATTC
Leptin	NM_013076.3	GGTTTCGTGGTGCTGACTAA	CACATCCTGTTCCGACTCTTAC
Adiponectin	BC092565.1	AAGTCTGGCTCCAAGTGTATG	GGTAGAGAAGGAAGCCTGTAAAT
β-actin	NM_031144.3	ACAGGATGCAGAAGGAGATTAC	ACAGTGAGGCCAGGATAGA

### Western blot determination of UCP-1 and β-actin

EFPs were homogenized in RIPA lysis buffer containing protease inhibitors (Sigma) for 30 min and then sonicated at 4°C. After centrifuged (16 000 × g for 10 minutes at 4°C), the supernatant was collected. The concentration of protein was measured by the Bradford method with a protein assay kit (Bio-Rad). The protein from the lysate was loaded at 40 μg, separated by 10% sodium dodecyl sulfate polyacrylamide gel electrophoresis, and then transferred onto nitrocellulose membranes (Millipore, USA). The blots were hybridized with primary antibodies against UCP-1 (Proteintech Group, USA) and β-actin (Cell Signalling Technology, USA). The horseradish peroxidase activity was detected by an enhanced chemiluminescence system (UVP BioSpectrum Imaging System, Upland, USA) following incubation with the horseradish peroxidase-conjugated secondary antibodies (Cell Signalling Technology). The values were obtained by dividing the density of the band of UCP-1 by that of β-actin from the same sample.

### Statistical analyses

Data were analyzed by a one-way analysis of variance (ANOVA) with Tukey’s post-hoc test. All results are presented as the mean ± standard error of the mean. p values < 0.05 were considered significant.

## Results

### Food intake, BW, and the feed conversion efficiency

**Figure 1.**
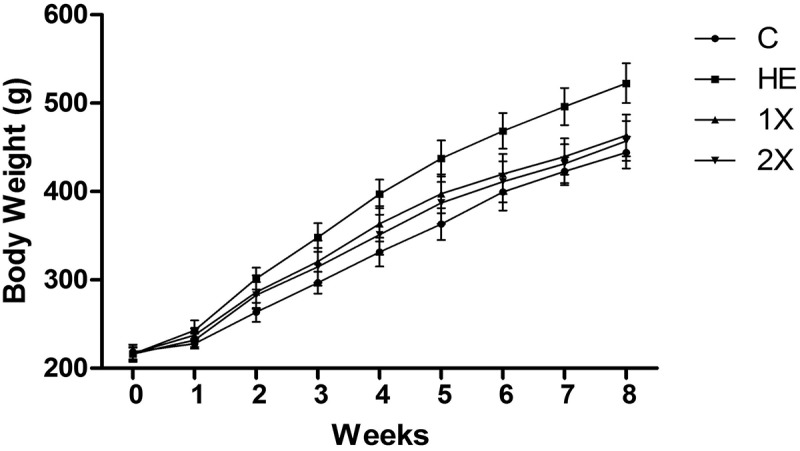
Body weights in eight weeks. C; vehicle control, HE; HED vehicle control, 1X; HED with 77.5 mg/kg/day of GTE, 2X; HED with 155 mg/kg/day of GTE. Data are the mean ± SEM (n  =  10 rats/group).

The effect of GTE on obesity was investigated using male SD rats with HED-induced obesity. Weekly food intake of each rat was approximately 140 ~ 173 g, and no difference was seen among all groups (Table 3). The average BW did not significantly differ between groups at week 0. From the 2nd week to the end of the trial, the HE group had significantly higher BW than the other groups. During the period of the study, average BW gains were 221.2 ± 17.7, 306.3 ± 19.6, 246.5 ± 26.4, and 241.0 ± 20.9 in the C, HE, 1X, and 2X groups, respectively (Figure 1). Although the weight of total food intake per rat was not different between the groups, the food conversion efficiency (Increase of BW / Total calories intake) was higher in the HE group than the C, 1X, and 2X groups (Table 3). The 1X group showed slightly higher BW and food conversion efficiency than the 2X group, but the difference was not statistically significant. Group C with the lowest average BW gain indicated that obesity successfully occurred in HED-fed rats.

**Table 3.** Effects of GTE on the body weight, diet intake, food efficiency, and serum biochemical parameters in HED-fed rats.

	C	HE	1X	2X
Total food intake (g)/rat	1386.7 ± 13.4^a^	1382.0 ± 18.9^a^	1377.9 ± 18.0^a^	1376.2 ± 19.1^a^
Increase of BW/rat	221.2 ± 17.7^a^	306.3 ± 19.6^c^	252.5 ± 24.7^b^	247.2 ± 16.6^b^
FCE (%)	15.9 ± 1.3^a^	22.2 ± 1.4^c^	18.4 ± 1.8^b^	17.9 ± 1.3^b^
TG (mg/dl)	44.3 ± 3.0^a^	79.0 ± 10.3^c^	55.1 ± 7.5^b^	52.1 ± 3.9^b^
TC (mg/dl)	42.0 ± 6.4^a^	69.5 ± 7.8^d^	61.2 ± 6.6 ^c^	53.3 ± 7.5 ^b^
LDL (mg/dl)	5.5 ± 0.7^a^	7.8 ± 1.2^b^	6.3 ± 1.6^a^	6.0 ± 0.9^a^
HDL (mg/dl)	20.1 ± 4.0^c^	13.4 ± 1.4^a^	16.0 ± 1.3^b^	16.1 ± 1.5^b^
FFA (μM)	3.2 ± 1.1^a^	4.9 ± 0.9^b^	3.9 ± 0.5^a^	3.5 ± 0.4^a^
AST (U/L)	126.9 ± 39.4^a^	224.1 ± 79.3^b^	171.9 ± 13.7^a^	163.1 ± 15.2^a^
ALT (U/L)	33.1 ± 4.6^a^	58.6 ± 9.7^c^	47.9 ± 6.7^b^	43.1 ± 11.6^b^

Control, vehicle control; HE, HED vehicle control; 1X, HED with 77.5 mg/kg/day of GTE; 2X, HED with 155 mg/kg/day of GTE. All values are the mean ± SEM (*n*  =  10 rats/group). Different superscript letters (a, b, c) indicate a significant difference at *p *< 0.05 by a one-way ANOVA with Tukey’s post-hoc test. BW, body weight; FCE, food conversion efficiency (Increase of BW/Total calories intake); TG, triacylglycerol; TC total cholesterol; FFA, free fatty acids; AST, aspartate transaminase; ALT, alanine transaminase.

### Plasma biochemical parameters

Plasma biochemistry was evaluated to assess the anti-obesity effects of GTE in HED-fed SD rats. Group C had lower TG, TC, LDL, FFA, AST, and ALT than the HE group, and lower TG, TC, and ALT than the 1X and 2X groups. The HDL level was highest in the C group (Table 3). GTE treatment resulted in significant decreases in TG, TC, LDL, FFA, AST, ALT, and FFA, and an increase of HDL in both the 1X and 2X groups compared to the HE group. Moreover, a lower concentration of TC was found in the 2X group than in the 1X group (Table 3).

### Fat accumulation and compositions of WAT

**Figure 2.**
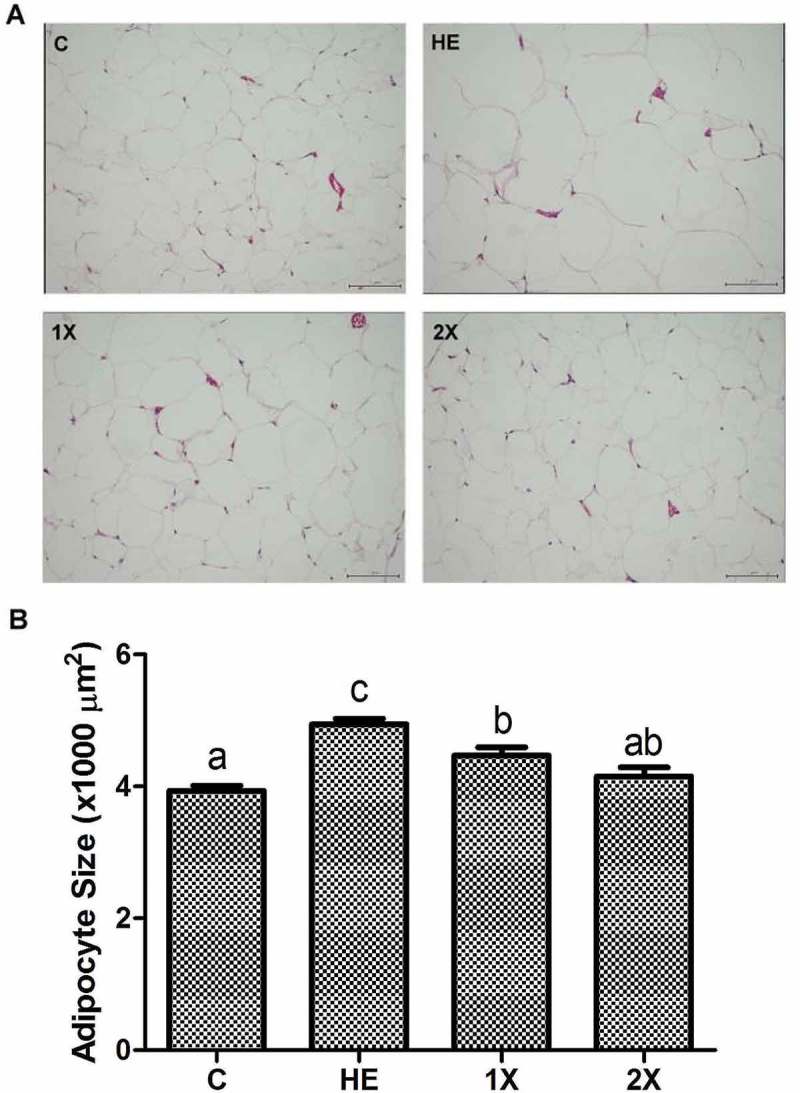
Histological analysis of epididymal fat pads stained with hematoxylin-eosin. C; vehicle control, HE; HED vehicle control, 1X; HED with 77.5 mg/kg/day of GTE, 2X; HED with 155 mg/kg/day of GTE. (a) Sections of epididymal fat pads fixed and stained with hematoxylin-eosin to visualize adipocytes (original magnification ×200). (b) Adipocytes were analyzed with an image analysis system and quantified. All values are the mean ± SEM (n  =  10 rats/group). Different superscript letters (a, b, c) indicate a significant difference at p < 0.05 by a one-way ANOVA with Tukey’s post-hoc test. Scale bar = 50μM.

Effects of GTE on fat accumulation were studied by analyzing the weight of WAT and the size of adipocytes. Weights of epididymal, perirenal, and mesenteric WATs were lower in the C, 1X, and 2X groups than in the HE group. Compared to the C group, the 1X group had higher weights of EFPs and renal fat pads (RFPs), and only RFPs were heavier in the 2X group. The body fat percentage of control rats was lower than those of HE and 1X rats, and both 1X and 2X rats had a lower body fat percentage than HE rats (Table 4). In addition, adipocytes of the EFPs were much smaller in rats of the 1X and 2X groups than in rats of the HE group (Figure 2).

**Table 4.** Effects of GTE on the mass of the EFP, RFP, MFP, and total body fat in HED-fed rats.

	C	HE	1X	2X
EFP (g)	6.6 ± 0.9^a^	12.4 ± 1.9^c^	8.9 ± 1.5^b^	7.5 ± 2.0^ab^
RFP (g)	10.9 ± 1.^a^	20.0 ± 1.7^c^	14.8 ± 2.2^b^	13.6 ± 1.8^b^
MFP (g)	5.6 ± 1.2^a^	9.7 ± 1.6^b^	6.9 ± 1.3^a^	6.3 ± 1.5^a^
Body fat percentage (%)	5.1 ± 0.6^a^	8.0 ± 0.9^c^	6.6 ± 0.8^b^	6.0 ± 0.8^ab^

Control, vehicle control; HE, HED vehicle control; 1X, HED with 77.5 mg/kg/day of GTE; 2X, HED with 155 mg/kg/day of GTE. All values are the mean ± SEM (*n*  =  10 rats/group). Different superscript letters (a, b, c) indicate a significant difference at *p* < 0.05 by a one-way ANOVA with Tukey’s post-hoc test.

### Leptin and adiponectin mRNA expressions

**Figure 3.**
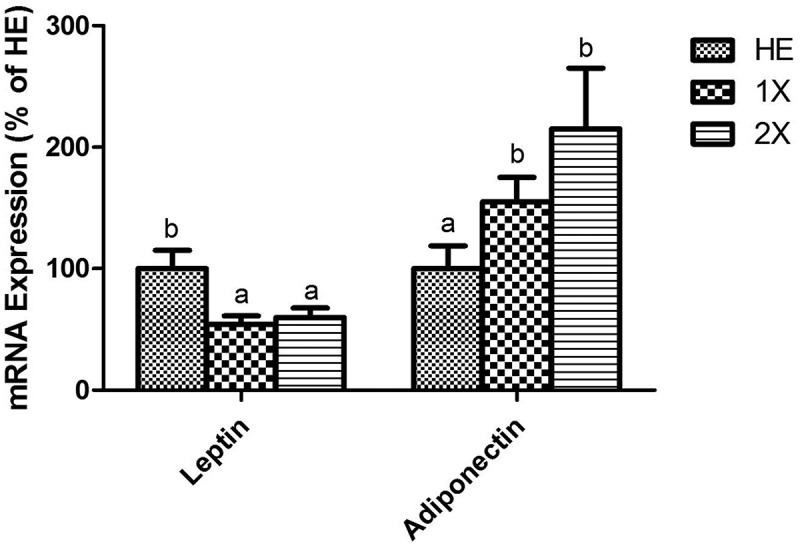
Expressions of leptin and adiponectin in the EFPs. HE; HED vehicle control, 1X; HED with 77.5 mg/kg/day of GTE, 2X; HED with 155 mg/kg/day of GTE. All values are the mean ± SEM (n  =  10 rats/group). Different superscript letters (a and b) indicate a significant difference at p < 0.05 by a one-way ANOVA with Tukey’s post-hoc test.

Since our purpose was to understand the anti-obesity mechanism of GTE in rats fed with a HED, the expressions of mRNA were compared in three HED fed groups. mRNA expressions of leptin and adiponectin were measured by an RT-qPCR. Leptin was higher in the HE group than in the 1X and 2X groups, and adiponectin was higher in the 1X and 2X groups than in the HE group. Although the 2X group showed higher expression of adiponectin than the 1X group, the difference was not significant (Figure 3).

### Expressions of beige-regulating and thermogenic genes in EFPs

**Figure 4.**
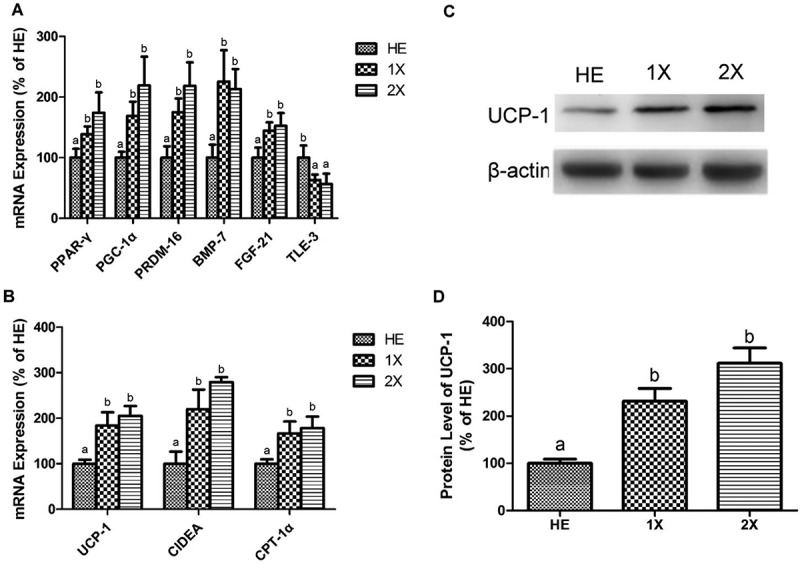
Expressions of genes related to beige transformation and thermogenesis in the EFPs. HE; HED vehicle control, 1X; HED with 77.5 mg/kg/day of GTE, 2X; HED with 155 mg/kg/day of GTE. Genes related to (a) beige transformation and (b) thermogenesis were detected by an RT-qPCR. Representative blots (c) and protein levels (d) of UCP-1 were detected using western blotting. All values are the mean ± SEM (n  =  10 rats/group). Different superscript letters (a and b) indicate a significant difference at p < 0.05 by a one-way ANOVA with Tukey’s post-hoc test.

Several genes that regulate the transformation of white into beige adipose tissue and thermogenesis were measured by an RT-qPCR to evaluate the effect of GTE on the acquisition of BAT and beige adipose tissue characteristics by white adipocytes. PPAR-γ, PRDM-16, BMP-7, FGF-21, and PGC-1α, which drive development of beige adipose tissue, were significantly higher in the 1X and 2X groups than in the HE group. Moreover, TLE-3, which suppresses brown-selective genes and induces white-selective genes, was downregulated in the 1X and 2X groups (Figure 4(a)). Consistently, the biomarkers of browning, including UCP-1, CPT-1, and CIDEA, were upregulated in the 1X and 2X groups. Expressions of these genes did not differ between the 1X and 2X groups (Figure 4(b)). Similar to the result of mRNA expression, the protein levels of UCP-1 were significantly higher in the GTE-treated groups than the HE group and were not different between the 1X and 2X groups (Figure 4(c,d)).

## Discussion

The anti-obesity mechanisms of GTE became an important topic in nutritional and food science research because beneficial effects of GTE against obesity were demonstrated in human and animal models [7,8,31]. In the present study, we provide evidence that GTE significantly improved obesity phenomena, such as BW, fat weight, the adipocyte size, and plasma chemical parameters in rats with HED-induced obesity. The reduction of leptin and induction of adiponectin in the study also indicated that GTE modulated obesity in rats. Moreover, genes upregulated in beige adipose tissue were higher in the 1X and 2X groups compared to the HE group; and TLE-3, a beige adipose tissue transformation suppressor [26], was downregulated in the 1X and 2X groups. To our knowledge, this is the first paper revealing that GTE alone can induce the biomarkers of beige adipose tissue and the multiple pathways of browning that involve either PPAR-γ, PRDM-16, FGF-21, BMP-7, or TLE-2 associated with a prevention of HED-induced obesity in SD rats.

Beige adipose tissue is thought to be a potential target of anti-obesity therapy due to its capacity for oxidizing lipids and dissipating energy [20,32]. In addition, the browning of WAT was reported to be highly correlated with the anti-obesity effect without an increase in BAT function. For example, transgenic expression of PRDM-16 in WAT promoted the development of beige adipose tissue and resistance to obesity without enhancing the BAT mass [33]. Moreover, UCP-1 overexpression in adipose tissue suppressed obesity [13]. Since PRDM-16 and UCP-1 are highly expressed in beige adipose tissue, an increase of beige adipose tissue should resist weight gain [23]. Therefore, determining the level of browning of WAT could help assess the anti-obesity effect of GTE. Browning is regulated by several pathways. PPAR-γ, PRDM-16, and FGF-21, belonging to two different pathways, induced PGC-1α to enhance expressions of genes involved in thermogenesis and fatty acid oxidation, such as UCP-1 [34], CIDEA [35,36], and CPT-1 [37,38]. Moreover, BMP-7 was a member of EWS/YBX-1/BMP-7 pathway, which drives browning by upregulating UCP-1 and downregulating the WAT-specific marker, TCF21 [39]. In contrast, TLE-3, an inhibitor of PRDM-16, suppresses the browning of WAT [26]. In the present study, GTE significantly induced PPAR-γ, PRDM-16, PGC-1α, BMP-7, and FGF-21, and reduced TLE-3, which was associated with enhancement of the browning biomarkers, UCP-1, CIDEA, and CPT-1, in the 1X and 2X groups compared to the HE group. These results revealed that WAT seemed to transform to beige adipose tissue within eight weeks when SD rats were given GTE daily. Sae-tan et al. [40] reported that a combination of decaffeinated green tea and voluntary exercise induced genes related to browning in mice fed high fat diets, but the browning effect was not observed when the mice were given only green tea [40]. Since exercise had already been linked to browning [41], the role of green tea in regulation of browning was still a puzzle. Moreover, they did not provided any physiological or weight data following treatments. Herein, we provide the first evidence that GTE alone was sufficient to induce browning-related genes in WAT, which was accompanied by a significant anti-obesity effect in HED-fed rats.

Browning is defined as a process that the expression of UCP-1 increases in WAT [26]. Browning has been observed in EFP and used for investigating browning in many previous studies [21,34,40,42–44]. Therefore, the gene expressions of EFP are undoubtedly important to evaluate if GTE induces browning in this study. Our results indicate that GTE could be able to induce browning, because the GTE up-regulated the biomarkers of beige and the pathways of browning in EFP. Since EFP was suggested to be least able to induce cold-derived browning [15] and to predominantly contain white adipocytes [41], browning may also occur in subcutaneous, renal, and mesenteric WATs post the treatment of GTE. However, more experiments are needed to understand the effect of GTE on browning in other WATs.

PPAR-γ has tissue-specific effects [45]. In adipocytes, PPAR-γ is known for its anti-obesity capability and insulin sensitization. PPAR-γ enhances fatty acid uptake and adipogenesis in WAT to reverse insulin resistance [46], but upregulation of PPAR-γ does not necessarily cause an increase in BW [47,48]. Moreover, Tian et al. [49] demonstrated that green tea polyphenols induced the extracellular signal-regulated kinase (ERK)1/2-PPAR-γ-adiponectin pathway followed by reduction of fat deposits [49]. Therefore, it is not surprising that a PPAR-γ agonist was reported to have anti-diabetes and anti-obesity capabilities [50]. For the anti-obesity effect, PPAR-γ can improve HED-induced obesity through browning of WAT and also upregulating adiponectin. Adiponectin is known to increase fatty acid oxidation and insulin sensitivity and prevent obesity and inflammation [51–53]. In the present study, we showed that both PPAR-γ and adiponectin increased following GTE treatment of HED-fed rats. Although the concentration of circulating adiponectin was not determined, an increase of circulating adiponectin was probable following GTE treatment due to the higher mRNA levels of adiponectin in WAT of the GTE-treated groups than the HE group. Circulating adiponectin is mainly secreted by adipocyte, and positive correlation was shown between the mRNA level of adiponectin and the concentration of circulating adiponectin [49,54–57]. Moreover, the increase of PPAR-γ mRNA in WAT was indicated to be associated with an increase of circulating adiponectin in the previous studies [49,58]. Thus, the results suggest that GTE might induce anti-obesity, anti-inflammatory, and insulin sensitization effects of adiponectin via upregulation of PPAR-γ in adipocytes. For detailing the systemic effect of GTE via adiponectin, a further study, such as to measure the concentration of circulating adiponectin, should be performed in the future.

In the present study, the C group was designed for evaluating if obesity was successfully induced by HED in the rats. Moreover, the results of the groups fed with a HED (HE, 1X, and 2X groups) were sufficient to support that GTE could induce browning-related genes and limit weight gain in HED-fed rats. Therefore, the gene expressions in WAT were not analyzed. Although gene expressions of the C group were not determined, GTE seemed to prevent the down-regulation of browning-related genes in HED-fed rats because lower mRNA expressions of browning-related genes in WAT were reported in the previous studies [40,59,60]. However, further study is necessary to investigate effects of GTE in the rats fed with different diets.

The anti-obesity effects of GTE are usually evaluated by physical differences in the bodies between rats given and those not given GTE. The GTE used in the current study significantly reduced increases in BW, WAT, and adipocyte size. The results indicated that obesity was modulated when HED-fed rats were given GTE. Furthermore, GTE-administered rats had lower levels of TG, TC, LDL, and FFA, and a higher level of HDL in serum. Increasing TG, TC, LDL, and FFA and decreasing HDL are frequently associated with cardiovascular disease, which is one of the obesity-related diseases. Since high serum FFA levels were observed in obese and diabetic patients [61] and promote hepatic steatosis, the GTE may prevent hepatic steatosis by reducing FFA levels. Preventing hepatic steatosis was also supported by our observation that GTE-administered rats showed lower serum ALT and AST levels which were correlated with hepatic steatosis and injury in obese mice [62]. Therefore, GTE could improve HED-induced obesity and might prevent its derivative diseases.

The effects of functional foods are influenced by the dose administered [63–69]. Overdosing results in toxicity [63,64], and an inadequate dose leads to low efficiency [65–69]. A high dose of GTE (EGCG > 100 mg/kg/day) increases serum ALT and AST levels due to induction of liver toxicity [64]. Since lower levels of ALT and AST were shown in the 1X and 2X groups compared to the HE group, neither dose of GTE used in this study appeared to cause liver injury. To understand the dose effect, we treated HED-fed rats with two different doses of GTE. Previous studies reported that receiving GTE at 750 mg/day significantly reduced the BW of human subjects [30,70]. According to a formula from the US Food and Drug Administration, 77.5 mg/kg/day of GTE for an SD rat is equivalent to 750 mg/day for a 60-kg human. Therefore, 77.5 and 155 mg/kg/day of GTE were used in the current study. Our results showed that when HED-fed rats were given 155 mg/kg/day of GTE (2X group), their BW, fat weight, plasma chemical parameters, and adipocyte size were slightly better than those of rats given 77.5 mg/kg/day of GTE (1X group); however, no statistical difference in these obesity parameters was shown between the 1X and 2X groups. Similarly, expressions of beige-related genes also are higher in the 2X group but did not statistically differ between the 1X and 2X groups. Therefore, 77.5 and 155 mg/kg/day of GTE seemed to be efficient doses for an anti-obesity effect, but we cannot exclude that the anti-obesity effects may be enhanced by a higher dose of GTE.

In summary, our study presents that GTE alone is sufficient to promote the biomarkers of beige adipose tissue and multiple pathways of browning in WAT and to safely improve HED-induced obesity in SD rats.
